# Highly flexible Ag nanowire network covered by a graphene oxide nanosheet for high-performance flexible electronics and anti-bacterial applications

**DOI:** 10.1080/14686996.2021.1963640

**Published:** 2021-09-15

**Authors:** Hyeong-Min Sim, Han-Ki Kim

**Affiliations:** School of Advanced Materials Science and Engineering, Sungkyunkwan University, Suwon-si, Republic of Korea

**Keywords:** Ag nanowire, graphene oxide, spray coating, transparent conductive electrode, thin film heater, electroluminescence, antibacterial, 20 Organic and soft materials (colloids, liquid crystals, gel, polymers), 103 Composites, 201 Electronics / Semiconductor / TCOs, 306 Thin film / Coatings

## Abstract

We investigated a flexible and transparent conductive electrode (FTCE) based on Ag nanowires (AgNWs) and a graphene oxide (GO) nanosheet and fabricated through a simple and cost-effective spray coating method. The AgNWs/GO hybrid FTCE was optimized by adjusting the nozzle-to-substrate distance, spray speed, compressor pressure, and volume of the GO solution. The optimal AgNWs/GO hybrid FTCE has a high transmittance of 88% at a wavelength of 550 nm and a low sheet resistance of 20 Ohm/square. We demonstrate the presence of the GO nanosheet on the AgNWs through Raman spectroscopy. Using scanning electron microscopy and atomic force microscopy, we confirmed that the nanosheet acted as a conducting bridge between AgNWs and improved the surface morphology and roughness of the electrode. Effective coverage by the GO sheet improved the conductivity of the AgNWs electrode Effective coverage of the GO sheet improved conductivity of the AgNWs electrode with minimum degradation of optical and mechanical properties. Flexible thin film heater (TFH) and electroluminescent (EL) devices fabricated on AgNWs/GO hybrid FTCEs showed better performance than devices on bare AgNWs electrodes due to lower sheet resistance and uniform conductivity. In addition, an AgNWs/GO electrode layer on a facial mask acts as a self-heating and antibacterial coating. A facial mask with an AgNWs/GO electrode showed a bacteriostatic reduction rate of 99.7 against *Staphylococcus aureus* and *Klebsiella pneumonia*.

## GRAPHICAL ABSTRACT

**Figure:**
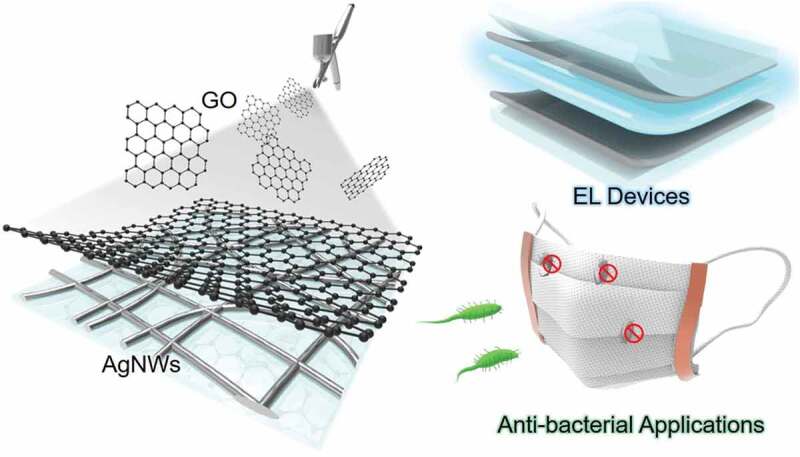

## 1. Introduction

A flexible and transparent conductive electrode (FTCE) is a thin film with high transparency, high conductivity, and outstanding flexibility [1–3]. Due to the rapid advancements in flexible electronics and wearable devices, FTCEs have gained a great deal of attention as a key component of flexible displays [4–6], flexible touch panels [7–9], flexible thin film heaters (TFHs) [10–16], photoelectric devices [17–20], and wearable electronics [21–23]. Currently, most flexible electronics and wearable devices use Sn-doped In_2_O_3_ (ITO) film coated on a flexible substrate through a typical magnetron sputtering process [24–26] owing to the high conductivity and transparency of commercially available ITO films. However, the brittle nature and poor mechanical properties of typical ITO films remain critical problems standing in the way of their use in FTCEs [27–31]. Furthermore, ITO electrodes are usually fabricated through an expensive vacuum process, such as evaporation or sputtering, and indium is very costly because reserves of the element are limited [32–34]. To address the problems of typical ITO electrodes, various substitutions, such as conductive polymers [35–37], carbon nanotubes [38,39], graphene [40,41], and metal nanowires [42,43], have been extensively investigated. Among several candidates, Ag nanowires (AgNWs) with a diameter of less than 100 nm and a length of several µm are thought to be promising materials to replace ITO films for fabrication of FTCE devices. An AgNWs film with an irregularly connected network has been applied in flexible electronics and wearable devices due to its high conductivity, low sheet resistance, good flexibility and simple solution-based coating process [44–46]. It is possible to produce a cost-effective AgNWs FTCE because it can be printed by spin coating [47,48], bar coating [49], slot die coating [7,50], brush painting [51], and spray coating [52] at room temperature. However, a reduction in the AgNW concentration in ink for the purpose of confirming high transmittance resulted in a reduction in the inter-link connectivity of the AgNWs and increased the sheet resistance. In addition, the high surface roughness and high contact resistance between AgNWs lead to leakage current and local heating of FTCEs. For those reasons, AgNWs still cannot replace commercial ITO film in the FCTE market. Graphene is also a core material for next-generation conductive transparent electrodes. Graphene exhibits a theoretical mobility of 200,000 cm^2^/V-s, and has high conductivity and excellent transparency due to its 2-dimensional (2D) structure [53]. In particular, its high Young’s modulus, which reaches about 1 TPa, enables outstanding mechanical flexibility [54]. However, the applicability of graphene electrodes in large-area product manufacturing is limited by the high cost of chemical vapor deposition and the complexity of the transfer process [55]. In order to solve these problems, research on graphene oxide (GO) nanosheets obtained through Hummers’ method has actively progressed [56]. Using Hummers’ method, although the sp^2^-hybrid bond of graphene is broken and shows high resistivity, a GO nanosheet can be obtained in large quantities as a stable solution. Moon et al. have demonstrated that spray coating of GO nanosheets leads to improvements in the surface roughness, haze, and conductivity of AgNWs [57]. They reported the feasibility of a GO nanosheet as an over-coating layer for AgNWs electrodes. However, they did not demonstrate the possible applications of GO over-coated AgNWs electrodes.

In this work, AgNWs and GO were dispersed separately in solution and then airbrushed onto a substrate to create a high-quality FTCE. Spray coating is a well-known method of solution coating in which functional ink is sprayed through a nozzle to deposit a film on a substrate. The spray coating process has a faster coating speed and easier operation than other solution process methods [58,59]. Optimization of the AgNWs/GO hybrid FTCE proceeded by maintaining the high transmittance of the AgNWs and improving the conductivity and surface properties of the electrode by controlling the spray volume of the GO solution. To illustrate the feasibility of the AgNWs/GO hybrid FTCE, we fabricated flexible TFHs, flexible electroluminescent (EL) devices, and an antibacterial facial mask. Successful operation of the flexible TFH and EL devices and antibacterial facial mask indicates that the AgNWs/GO hybrid electrode is a promising substitute for ITO electrodes and solves the critical problems associated with both AgNWs and GO nanosheets.

## 2. Experimental procedures

### 2.1. Spray coating of AgNWs/GO hybrid FTCEs

**Figure 1.**
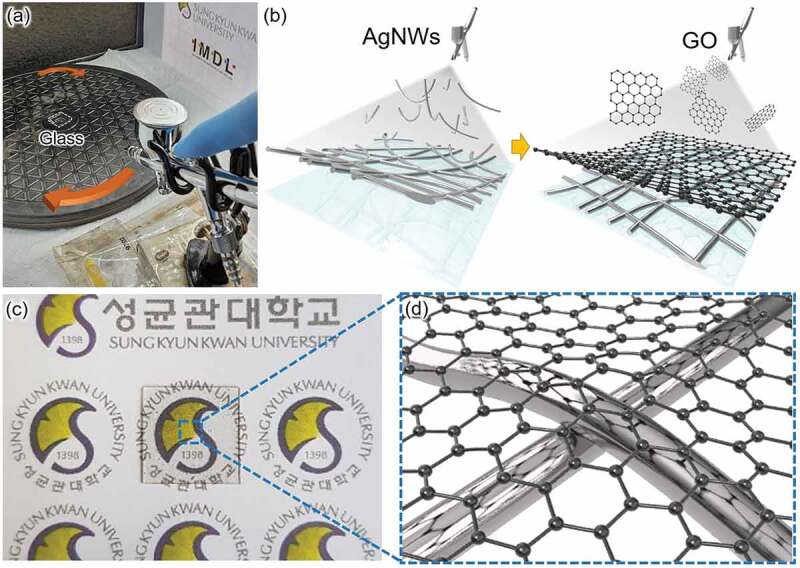
(a) Photograph of the lab-made spray coating system for AgNWs and GO coating. (b) Schematic diagram of the spraying process used to fabricate the AgNWs/GO FTCE. (c) Photograph of the AgNWs/GO FTCE electrode coating on the PET substrate and (d) schematic illustration of enlarged surface showing the contact region between the AgNWs and GO nanosheet

As shown in Figure 1(a,b), the hybrid FTCE electrode was fabricated by spraying a AgNWs solution (length, 25 µm; diameter, 25 nm; Flexio Co. Ltd) and a GO nanosheet solution (multilayer, Sigma-Aldrich) onto a substrate (1.5 ×1.5 cm^2^) placed on a rotating platform using a lab-designed airbrush coating system (PS-289, Mr.HOBBY). The AgNWs solution, consisting of 0.125 wt% AgNWs, was dispersed in an isopropyl alcohol solvent and then spray coated onto the substrate. The substrate was allowed to dry at 70°C for 5 minutes on a hot plate. This AgNWs coating process was repeated 3 times. Then, the GO solution was developed by dispersing 0.1 wt% GO in an isopropyl alcohol solvent in a sonicator for 60 minutes. GO solution was spray coated on top of the AgNWs-coated substrate as same spraying condition with AgNWs solution, which was then dried on the hot plate for 5 minutes at 70°C. The transmittance of the optimized AgNWs/GO hybrid FTCE was measured using a UV-vis spectrometer (V-670, Jasco) and the sheet resistance was measured through the four-point probe method (FPP-HS 8, DASOLENG Co. Ltd). The surface morphology and roughness of the optimized FTCEs was analyzed using field emission secondary electron microscopy (FE-SEM; JSM-7600 F, JEOL) and atomic force microscopy (AFM; Nanowizard Ultra Speed, JPK). A micro-Raman spectrometer system (Witec, Alpha 300 M+) was used to analyze the structure of the AgNWs/GO FTCE and confirm the presence of GO.

### 2.2. Fabrication of flexible and transparent TFHs

The optimized AgNWs/GO hybrid FTCE was used as an electrode for a TFH device. On the spray coated AgNWs/GO hybrid FTCE, an Ag interconnector was sputtered to lower the contact resistance when a direct current (DC) voltage was applied to the electrode. To compare the performance of TFHs with an AgNWs/GO hybrid electrode vs. bare AgNWs electrode, a reference TFH using AgNWs was fabricated under the same conditions. Heat was generated by applying DC voltage to both sides of the TFH and measured by a thermocouple at the center. Also, the change in temperature of the TFH following application of DC voltage was monitored with an infrared (IR) camera.

### 2.3. Fabrication of flexible EL devices

An EL prepolymer was prepared by mixing ZnS:Cu phosphors (blue color, GG 65) and liquid polydimethylsiloxane in a ratio of 2:1. The flexible EL device was fabricated by spin coating the EL prepolymer on the AgNWs/GO FTCE as the bottom electrode, and contacting another AgNWs/GO FTCE as the top electrode on the EL layer. The fabricated EL device was cured in an oven at 80°C for 1.5 hours. As a reference sample, an EL device with the same structure was fabricated using a single AgNWs electrode. Alternating current (AC) voltage was applied to each sample using a function generator connected to a power amplifier. The luminance according to the applied AC voltage was measured through a spectroradiometer (CS-2000, Konica Minolta).

### 2.4. Fabrication of self-heating antibacterial facial mask

The optimized AgNWs/GO hybrid solution was deposited onto the outer surface of the mask to create a self-heating antibacterial mask. In order to check the heat generation characteristics of the fabricated mask, a sample was prepared by attaching Cu tape on both sides of a mask coated with AgNWs/GO hybrid nanoparticles on its outer surface. Then, DC voltage was applied to both sides and the successful operation of the self-heating antibacterial facial mask was visually confirmed through the IR camera. Antibacterial tests were conducted to assess the antibacterial properties of the AgNWs. The KSK 0693 test kit was used to test the self-heating antibacterial mask. Using the kit, the same number of *Staphylococcus aureus* and *Klebsiella pneumonia* microbes were incubated with both the normal mask and the antibacterial mask for 18 hours at 37°C.

## 3. Results and discussion

**Figure 2.**
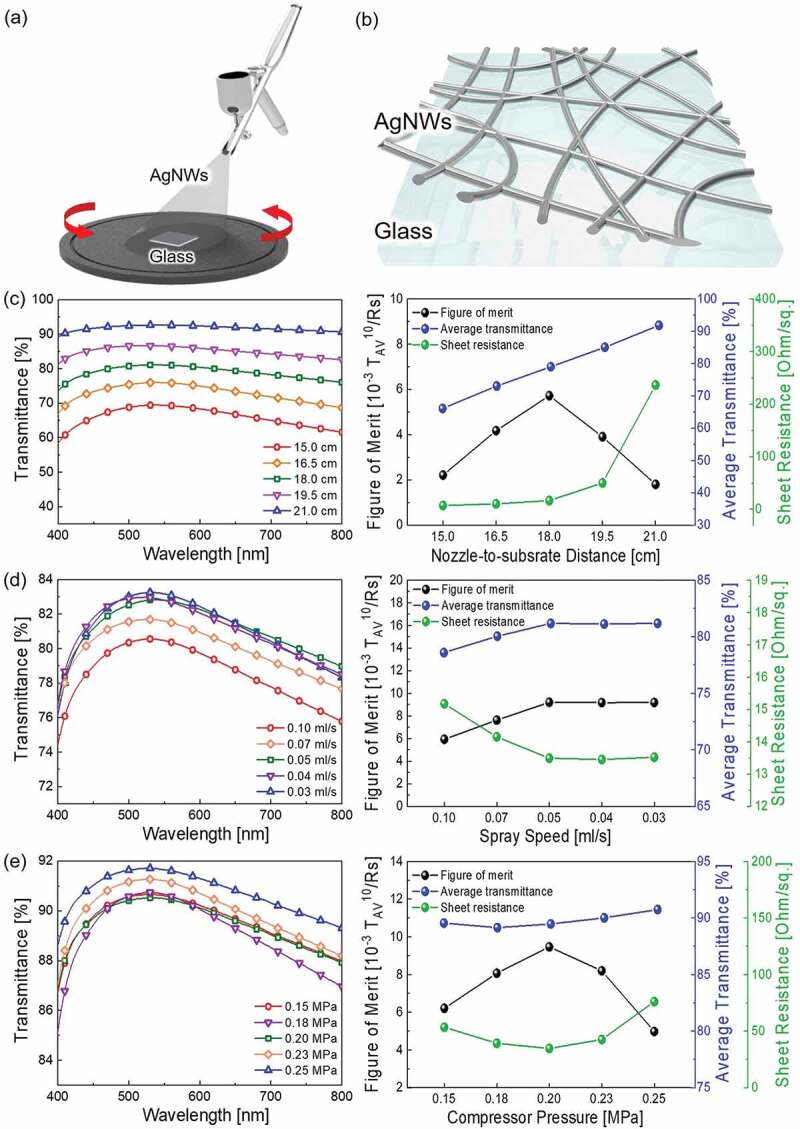
(a) Schematic illustration of the AgNWs solution spray coating process and (b) structure of the AgNWs TCE. Optical transmittance and FoM value of spray coated AgNWs as a function of nozzle-to-substrate distance (c), spraying speed (d), and compressor pressure (e)

Figure 2(a,b) show schematic diagrams of the AgNWs TCE fabrication process and the structure of the manufactured AgNWs TCE. In general, important parameters which have been extensively studied for spray coating using an airbrush are nozzle-to-substrate distance, compressor pressure, spray speed, substrate temperature, nozzle diameter and concentration of solution [60–62]. However, the spray speed of the solute is determined by solution concentration and nozzle diameter [63]. Accordingly, the spray speed was accurately controlled by calculating the amount of solution sprayed per unit time by finely adjusting the nozzle valve at a constant solution concentration. Therefore, the electrical and optical properties of AgNWs TCEs were optimized by nozzle-to-substrate distance, compressor pressure, spray speed and substrate temperature. In particular, substrate temperature was applied at a low temperature of 70°C between processes to ensure that the solvent was evaporated without causing deformation to the flexible polymer substrate. In consequence, the AgNWs TCE was optimized through measurement of the transmittance, sheet resistance, and figure of merit (FoM) values according to changes in the nozzle-to-substrate distance, spray speed, and compressor pressure, which have a major effect on the reproducibility and properties of the electrode. The FoM is defined by the equation below. The higher the FoM value, the better the performance of the TCE [64].

(1) $$FoM=10^{3}\frac{{T_{av}}^{10}}{R_{s}}$$

Here, *T_av_* is average transmittance and *R_s_* is sheet resistance. In the process of optimizing the nozzle-to-substrate distance, the spray speed was fixed at 0.10 ml/s and the compressor pressure was fixed at 0.20 MPa. As shown in Figure 2(c) and Table 1, the AgNWs TCE created with a spray nozzle-to-substrate distance of 18.0 cm showed high conductivity and optical transmittance. When the spraying distance is too short, the solution cannot be spread sufficiently and the transmittance decreases due to excessive solution deposition. Conversely, when the spraying distance is too long, the solution spreads well, but the amount of solution that ends up coated on the substrate decreases in comparison to the amount of sprayed solution. So, when the distance between the sample and the nozzle was 18.0 cm, the highest FoM value was obtained due to the high optical transmittance and low sheet resistance. Figure 2(d) and Table 2 show the electrical and optical properties of the spray coated AgNWs TCE according to spraying speed when the nozzle-to-substrate distance and compressor pressure were fixed at 18.0 cm and 0.20 MPa. The spray speed is determined by the amount of a solution sprayed per unit time at a constant air pressure. Sufficient time is required for the solvent to evaporate. Thus, it is essential to control the spray speed in order to find a compromise between the process efficiency that can be obtained by spraying the solution quickly and the quality of the electrode that can be obtained by spraying slowly so that the solvent can evaporate enough. When the spraying speed of the solution is too fast (above 0.10 ml/s), droplets form before the solution dries. As a consequence, solute does not spread uniformly and aggregates, causing a decrease in transmittance. When the spraying speed decreased to 0.05 ml/s, since the solvent had enough time to evaporate, creation of a uniform coating was possible without agglomeration of solution on the substrate, so both transmittance and sheet resistance improved. Even when the spraying speed decreased to 0.03 ml/s, there was no significant change in electrical and optical properties. Thus, the appropriate spraying speed was determined to be 0.05 ml/s in consideration of the efficiency of the process. During the optimization of the compressor pressure, the nozzle-to-substrate distance and the spray speed were fixed at 18.0 cm and 0.05 ml/s. As shown in Figure 2(e) and Table 3, with low compressor pressure, the kinetic energy of the sprayed solution is insufficient, making it difficult to form a uniform AgNWs network. Therefore, the AgNWs TCE showed high sheet resistance. Conversely, when the pressure of the compressor is too high, the solution on the substrate is blown out and the sheet resistance increases. So, when the spray coating was applied with a compressor pressure of 0.20 MPa, the resulting AgNWs TCE showed the highest FoM value. Based on three important spraying parameters, the optimum spraying conditions were determined to be: a compressor pressure of 0.20 MPa, spraying speed of 0.05 ml/s and spraying distance of 18.0 cm.

**Table 1.** Electrical and optical properties of the AgNWs TCE according to nozzle-to-substrate distance

Distance [cm]	Average transmittance [%]	Transmittance at 550 nm [%]	Sheet resistance [Ohm/square]	FoM [10^−3^Ω^−1^]
15.0	66	69	7	2.2
16.5	73	76	10	4.2
18.0	79	81	16	5.7
19.5	85	93	51	3.8
21.0	92	93	236	1.8

**Table 2.** Electrical and optical properties of the AgNWs TCE according to spraying speed

Speed [ml/s]	Average transmittance [%]	Transmittance at 550 nm [%]	Sheet resistance [Ohm/square]	FoM [10^−3^Ω^−1^]
0.10	79	81	15	5.9
0.07	80	82	14	7.6
0.05	81	83	14	9.2
0.04	81	83	14	9.2
0.03	81	83	14	9.2

**Table 3.** Electrical and optical properties of the AgNWs TCE according to compressor pressure

Pressure [MPa]	Average transmittance [%]	Transmittance at 550 nm [%]	Sheet resistance [Ohm/square]	FoM [10^−3^Ω^−1^]
0.15	90	91	53	6.2
0.18	89	92	39	8.1
0.20	90	91	35	9.5
0.23	90	91	43	8.2
0.25	91	92	76	5.0

**Figure 3.**
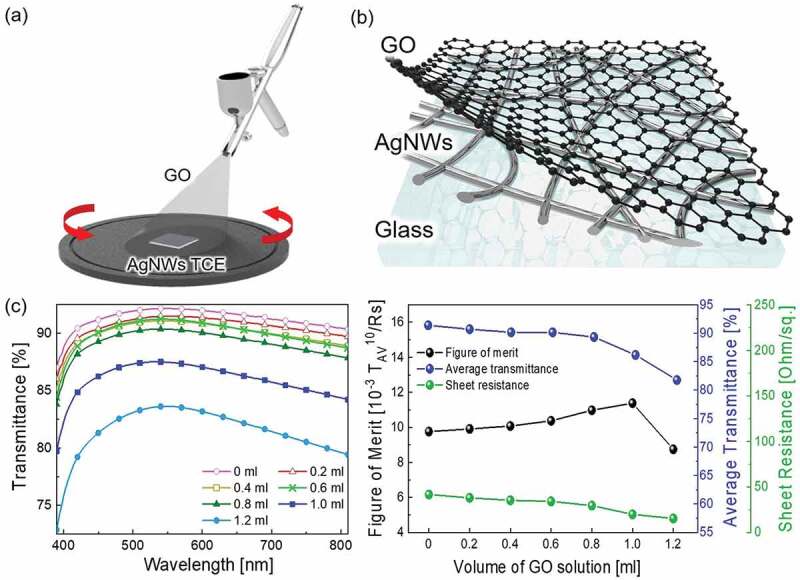
(a) Schematic diagram of spray coating of GO solution on AgNWs and (b) structure of the AgNWs/GO FTCE. (c) Transmittance according to the volume of sprayed GO solution and FoM values calculated from the average transmittance and sheet resistance

After optimization of the AgNW network, the GO nanosheet was sprayed on the AgNW network electrode. Schematic illustrations of the AgNWs/GO FTCE fabrication process and structure are shown in Figure 3(a–c) and Table 4 exhibit the electrical and optical properties according to the amount of GO solution sprayed onto the optimized AgNWs electrode. In the optimal coating process for the AgNWs TCE, the nozzle-to-substrate distance, spray speed, and compressor pressure were adjusted to avoid agglomeration of the solution and improve uniformity. Because both the GO solution and the AgNWs solution have similarly low solute concentrations and the same isopropanol solvent, there is no significant difference in viscosity or volatility (see supplementary materials 1). Through the FE-SEM analysis (Figure S1), it was confirmed that GO was uniformly deposited without non-uniform parts due to droplet formation when the GO solution was sprayed under the same conditions as the optimal AgNWs TCE spraying conditions. Subsequently, to control only the amount of GO deposited to reach the optimum FoM value while the parameters that ensure uniformity are fixed, the volume of the GO solution was adjusted as a new parameter. Notably, the sheet resistance decreased slightly when the GO solution was sprayed on the AgNWs electrode. In particular, when 1.0 ml of GO solution was sprayed, the AgNWs/GO hybrid electrode showed a sheet resistance of 20 Ohm/square and optical transmittance of 86%, which could make it applicable to flexible TFHs and EL devices. When more GO solution was sprayed, the sheet resistance decreased very slightly, but the transmittance further decreased, so the FoM rapidly decreased. Therefore, the optimal AgNWs/GO hybrid FTCE was produced by spraying of 1.0 ml of GO solution.

**Table 4.** Electrical and optical properties of the AgNWs/GO FTCE according to the spraying volume of GO solution

GO [ml]	Average transmittance [%]	Transmittance at 550 nm [%]	Sheet resistance [Ohm/square]	FoM [10^−3^Ω^−1^]
0	91	92	42	9.8
0.2	91	92	38	9.9
0.4	90	91	35	10.1
0.6	90	91	34	10.4
0.8	89	90	30	11.0
1.0	86	88	20	11.4
1.2	82	84	15	8.8

**Figure 4.**
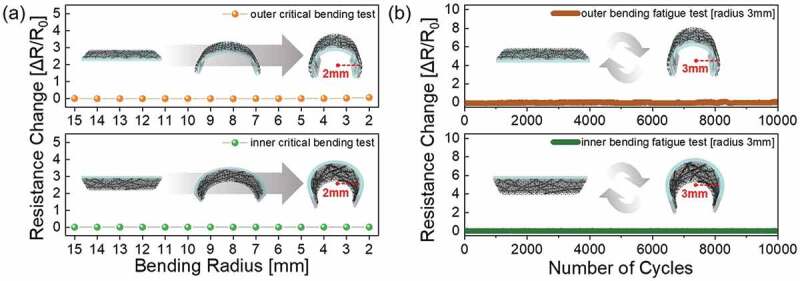
(a) Outer/inner bending test of the AgNWs/GO FTCE spray coated on PET substrate as a function of bending radius. (b) Outer/inner bending fatigue test of AgNWs/GO FTCE repeated 10,000 times at a 3 mm bending radius

To demonstrate the flexibility of the AgNWs/GO FTCE, we measured the change in resistance as the bending radius decreased. Figure 4(a) shows the change in resistance during the inner and outer bending test for the AgNWs/GO FTCE. In general, application of tensile stress (outer bending) to an FTCE causes a greater change in resistance than application of compressive stress (inner bending) [65]. However, the AgNWs/GO FTCE showed no difference in resistance regardless of the bending mode (inner vs. outer bending). Even at an inner and outer bending radius of 2 mm, the AgNWs/GO FTCE shows no resistance change, indicating excellent flexibility. Due to the limits of our bending test system, a further decrease in the inner bending radius of the AgNWs/GO FTCE was not possible. Figure 4(b) shows the dynamic bending test results for the AgNWs/GO FTCE. At a fixed bending radius of 3 mm, the AgNWs/GO FTCEs were subjected to 10,000 cycles of inner and outer bending. Following repeated inner and outer bending, we observed no change in resistance due to the outstanding flexibility of the AgNWs/GO FTCEs. Based on the results of the mechanical tests, the AgNWs/GO FTCE has sufficient mechanical flexibility for applications in flexible electronics without degrading the mechanical properties of AgNWs TCE (Figure S2).

**Figure 5.**
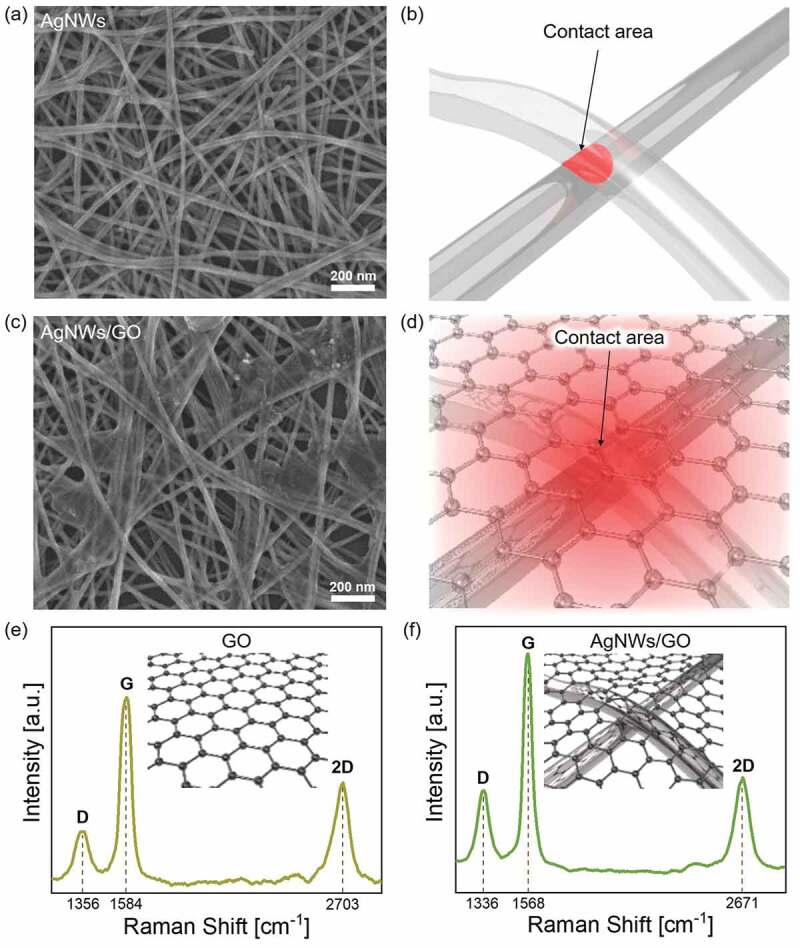
(a) FE-SEM image of the AgNWs TCE with (b) schematic illustration of contact area between AgNWs. (c) FE-SEM image of the AgNWs/GO FTCE with (d) schematic illustration of contact area between AgNWs under the overcoated GO nanosheet. Raman spectra of (e) GO and (f) AgNWs/GO FTCE

Figure 5(a,c) and Figure S1 show surface FE-SEM images of AgNWs and the optimized AgNWs/GO FTCE. In the case of bare AgNWs, the FE-SEM image showed a percolating AgNW network structure, as in previous reports [66–68]. The randomly percolating network provides a conduction path for current. Figure 5(b) illustrates the small contact region between AgNWs. However, in the case of the AgNWs/GO hybrid electrode, GO covered the AgNWs network and acted as a conductive bridge between AgNWs. As illustrated in Figure 5(d), the GO layer helps to decrease the high contact resistance caused by the small contact area of AgNWs by covering the disconnected part of the network, thus enhancing the electrical conductivity. In addition, if there is excessive deposition of the relatively low-conductivity GO layer, the excess results in a loss of optical transmittance.

Figure 5(e,f) and show the peak shifts of Raman spectra of the bare GO and optimized AgNWs/GO FTCE. Both samples showed clear D, G, and 2D peaks, indicating the presence of GO on AgNW network. D peak is a peak activated by a structural defect in GO. The G peak is a peak generated by the first order double-generated scattering process due to the E_2g_ symmetry of the sp^2^ carbon lattice. The 2D peak is a peak arisen by the second-order phonon mode between two in-plane transverse optical phonons [57,69–71]. Studies on the interaction of Raman effect occurring in the hybrid system of silver nanostructures and graphene have been extensively reported [72–74], and AgNWs are known to have more influence on mechanical strain than charge transfer to graphene [75]. Unlike graphene deposited on a flat substrate, the graphene deposited on a rough surface such as Ag NWs network suffers tensile stress by the curvature of AgNWs. As shown in graphs, when GO was deposited on AgNWs, the D and G bands redshifted by 20 and 24 cm^−1^. Especially the 2D band, which is most sensitive to the strain effect [76], showed a redshift of 32 cm^−1^. The shift rate of the Raman D, G, 2D band position ($\omega_{p}$) with strain (ε) applied to graphene can be expressed by the following two equations [76,77].

(2) $$\Delta \omega_{p}=-\gamma_{p}^{uniax}\omega_{p}^{0}\left(1-\nu \right)\varepsilon$$

$\gamma_{p}^{uniax}$ is the Grüneisen parameter under uniaxial strain for each Raman band, and $\omega_{p}^{0}$ is the Raman band position at zero strain, which are all positive value. ν is the Poisson’s ratio of the substrate. $\omega_{G^{+}}$ and $\omega_{G^{-}}$ were not considered because the uniaxial strain caused by the AgNWs was not high enough to separate the G peak into G^−^ and G^+^ peak [77].

(3) $$\Delta \omega_{p}=-\gamma_{p}^{biax}\omega_{p}^{0}\left(\varepsilon_{ll}+\varepsilon_{tt}\right)$$

$\gamma_{p}^{biax}$ represents the Grüneisen parameter during biaxial strain for each Raman band and $\varepsilon_{ll}$ and $\varepsilon_{tt}$ are hydrostatic component of the applied strain with the directions parallel (l) and perpendicular (t), all of which are positive numbers. Since the right side of both Equations are always negative value, redshift occurs in the D, G, and 2D peaks of strained GO [78–80]. Through the redshift of the Raman peak, it is clear that GO on the AgNWs under the tensile stress. According to the result of AFM analysis in Figure S3, the surface roughness of AgNWs/GO FTCE was improved from 17.80 nm to 8.89 nm compared to AgNWs TCE. The reason for the diminished surface roughness is that GO effectively improved the surface morphology by filling the empty spaces which is the cause of increasing roughness between AgNWs. These analyses confirmed that AgNWs were well-coated with GO, and show that GO helps to enhance the electrical and surface properties of AgNWs by improving the connectivity of AgNWs.

**Figure 6.**
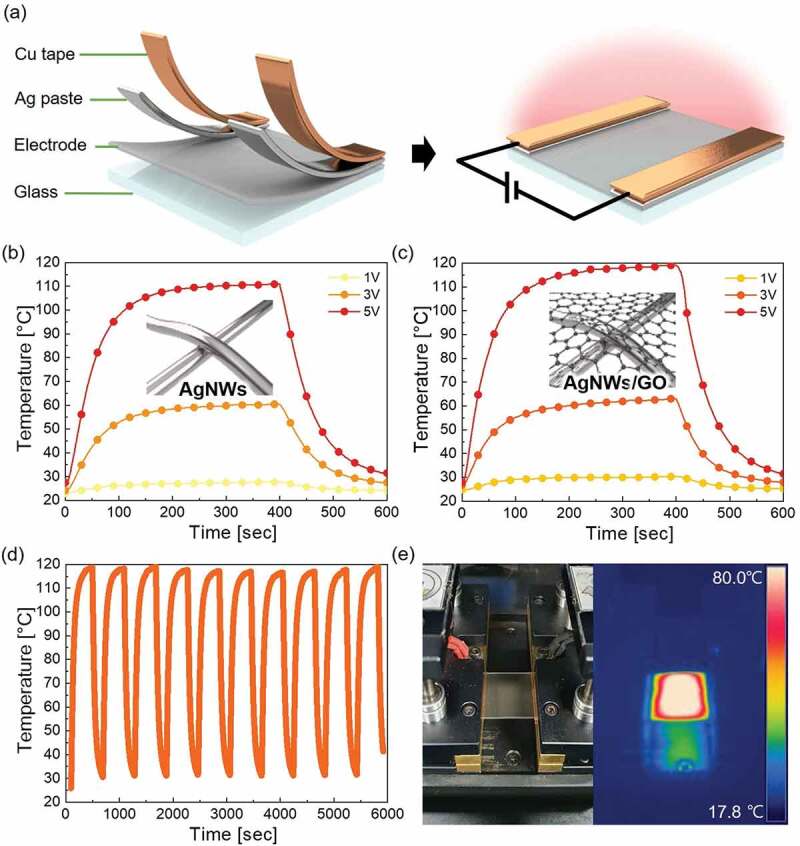
(a) Schematic illustration of the structure of the AgNWs/GO FTCE-based TFH. Temperature change over time when DC voltage is applied to the TFH with (b) AgNWs TCE and (c) AgNWs/GO FTCE. (d) Thermal stability of the TFH with AgNWs/GO FTCE. (e) Photograph and IR image showing heating of the TFH with AgNWs/GO FTCE when power is applied to the electrode

Figure 6(a) illustrates the structure of the TFH made of an AgNWs/GO FTCE and AgNWs TCE. Silver paste was applied to both sides of the AgNWs/GO FTCE, and Cu tape was attached to the paste to serve as a contact electrode. The TFH was operated by connecting the power supply and applying DC voltage. Figure 6(b,c) show the change in temperature over time when DC voltages of 1, 3, and 5 V were applied to each of the TFHs. DC voltage was applied to the electrode for 400 seconds, followed by a 200 seconds cooling period. Since AgNWs generate more Joule heating per unit area than ITO, carbon nanotube, graphene, etc., they produce more heat with less voltage and show excellent performance as TFHs [81]. The TFH with the AgNWs TCE showed a saturation temperature of 111°C at a voltage of 5 V, while the TFH using AgNWs/GO TCE showed a saturation temperature of 119°C at a voltage of 5 V; based on Joule’s law, the relationship between sheet resistance of TFH (*R*) and the generated temperature (*ΔQ_g_*) can be expressed as equation

(4) $$\Delta Q_{s}=\frac{V^{2}}{R}\Delta t=Q_{conv}=h_{conv}A_{conv}\left(T_{s}-T_{i}\right)$$

where *V* is input voltage and *t* is heating time; *Q_conv_* is heat loss due to convection in air; *h_conv_, A_conv_, T_s_*, and *T_i_* are convective heat transfer coefficient, surface area, saturation temperature, and initial temperature, respectively [82,83]. In this experiment, the heating time and applied voltage for each TFH are the same. From this equation, it is clear that the saturation temperature of TFH is inversely proportional to the sheet resistance of TFH. Since the sheet resistance of AgNWs/GO FTCE is lower than AgNWs TCE, AgNWs/GO FTCE-based TFH showed better performance than AgNWs TCE-based TFH. Spraying of the GO solution improved the connectivity and surface morphology of the AgNWs network. Therefore, spraying of GO on AgNWs helps to improve the performance of AgNWs-based TFHs. In general, the non-uniform distribution of AgNWs in a TFH leads to Joule heating at the narrow contact area between the nanowires due to the localized current density, which results in the failure of the nanowire network. However, as shown in Figure 6(d), even in the heat stability test, which was repeated 10 times under the same conditions, the FTCE showed excellent stability without any change in heating performance. The IR image of the TFH during operation in Figure 6(e) allows for visual observation of uniform heat generation due to the excellent characteristics of the AgNWs/GO FTCE.

**Figure 7.**
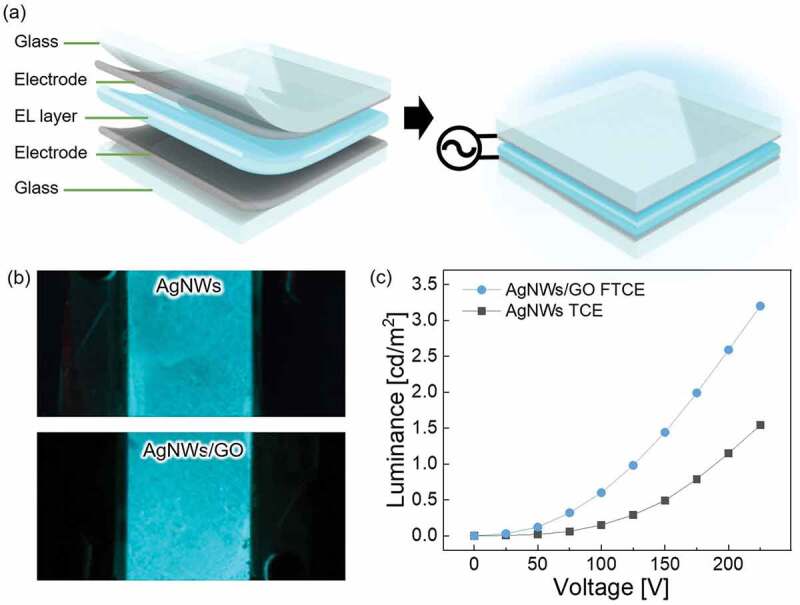
(a) Schematic structure of EL devices with the AgNWs/GO FTCE. (b) Photographs of a blue-emitting EL cell with AgNWs TCE and AgNWs/GO FTCE when the same AC voltage is applied. (c) Changes in luminance of the EL cell with AgNWs TCE and EL cell with AgNWs/GO FTCE according to AC voltage

Figure 7(a) shows the structure of the EL devices with the AgNWs TCE and AgNWs/GO hybrid FTCE. The EL layer was coated between the top and bottom electrode and AC voltage was applied to each electrode. Figure 7(b) shows photographs of the EL devices with the AgNWs TCE and AgNWs/GO FTCE at an AC voltage of 200 V, and Figure 7(c) shows the change in luminance when 0 to 225 V was applied to each device. The relationship between AC voltage and luminance is as follows [84].

(5) $$L=L_{0}exp\left(-\frac{\beta }{V^{\frac{1}{2}}}\right)$$

Here, *L* is the luminance, *V* is the applied voltage, and *L_0_* and *β* are constants determined by the device. As shown in Figure 7(c), each sample shows a sharp increase in luminance as the applied voltage increases, and the EL device with the GO layer on top of the AgNWs shows higher luminance when the voltage is applied due to the higher conductivity and reduced surface roughness of the electrode.

**Figure 8.**
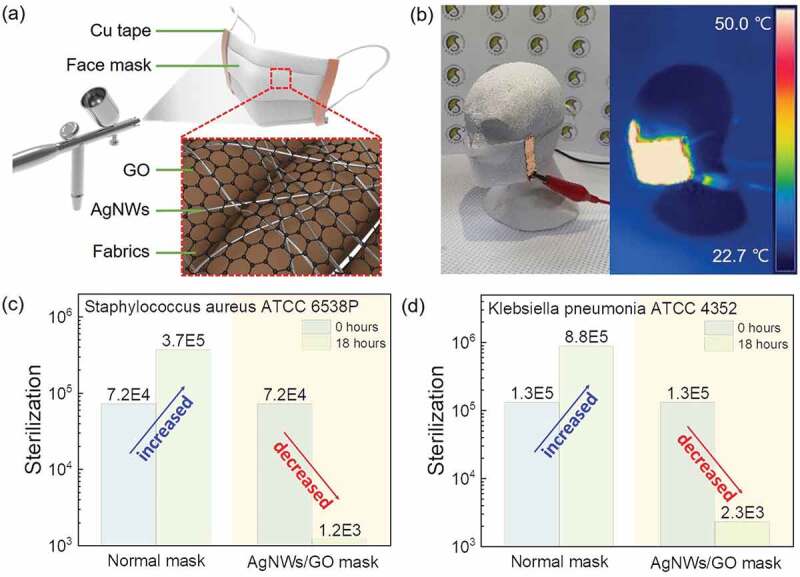
(a) Schematic illustration of the structure of the self-heating and antibacterial mask using AgNWs/GO FTCE. (b) Photograph and IR image of the self-heating and antibacterial mask in operation. (c) Number of initial bacteria vs. number of bacteria after 18 hours on the normal mask and AgNWs/GO mask incubated with *Staphylococcus aureus*. (d) Number of initial bacteria vs. number of bacteria after 18 hours on the normal mask and AgNWs/GO mask incubated with *Klebsiella pneumonia.*

The optimized AgNWs/GO FTCE was coated on the facial mask to assess the efficiency of its antibacterial effect and demonstrate stable heat generation under application of DC voltage. Figure 8(a) shows a schematic diagram of the fabrication process and surface structure of the self-heating antibacterial mask with the optimized AgNWs/GO FTCE. The AgNWs/GO FTCE was spray coated on the surface of the mask filter. Cu tape was attached to both ends of the mask to serve as a contact electrode for application of DC voltage. Figure 8(b) and supplementary material 2 shows an IR image and video of successful heat generation by the self-heating antibacterial facial mask. Even on a curved sculpture similar to a human face, the self-heating antibacterial mask with the AgNWs/GO FTCE showed uniform heat generation over the entire mask surface. This photograph and Figure 4 demonstrate that the AgNWs/GO FTCE deposited on the mask maintains stable performance even under the bending that occurs when the mask is actually worn on the face based on its excellent mechanical and electrical properties. The mechanism for the antibacterial action of silver has not yet been clearly elucidated, though many studies have been conducted over the past 60 years in attempts to clarify this issue [85,86]. One of the most convincing potential mechanisms holds that silver ions released from the surface of silver damage the structure of bacterial cell membranes and inhibit the activity of membranous enzymes [87]. Several studies have addressed the change in antimicrobial activity according to the surface area of silver, which showed that nanometer-sized silver with an extremely wide surface area allows for better contact with microorganisms and thus excellent antibacterial activity [88–90]. AgNWs, which have nanometer-sized wires, also have very large surface areas. An antibacterial test was conducted to assess whether the excellent antibacterial activity of AgNWs was maintained even in the mask with the AgNWs/GO FTCE coating on its surface. Figure 8(c,d) shows the results of the antibacterial test for a self-heating antibacterial mask, which was conducted using the KSK 0693 kit. This test method measures the number of bacteria that remain after 18 hours following inoculation of antibacterial fibers and ordinary fibers with the same number of bacteria. Figure 8(c) shows the number of *Staphylococcus aureus* microbes that remained 18 hours after 7.2 × 10^4^ bacteria were applied to a mask coated with the AgNWs/GO FTCE and an uncoated mask. After 18 hours, the number of bacteria on the normal mask increased to 3.7 × 10^5^, while the number of bacteria on the antibacterial mask decreased to 1.2 × 10^3^. These data indicate that the self-heating antibacterial mask has good antibacterial properties, as it was associated with a bacteriostatic reduction rate of 99.7 according to the following equation.

(6) $$R_{bac}=\frac{N_{nor}-N_{ant}}{N_{nor}}$$

Here, *R_bac_* indicates a bacteriostatic reduction rate, *N_nor_* indicates the number of viable bacteria after 18 hours on the normal mask and *N_ant_* indicates the number of viable bacteria after 18 hours on the antibacterial mask. Similarly, Figure 8(d) shows the number of bacteria remaining on each mask after an 18-hour incubation with 1.3 × 10^5^ *Klebsiella pneumonia* bacterial cells. After 18 hours, the normal mask showed 8.8 × 10^5^ bacteria, while the antibacterial mask showed just 2.3 × 10^3^, for a bacteriostatic reduction rate of 99.7. The results of the antibacterial tests using both bacteria indicate that the self-heating antibacterial mask exhibits excellent antibacterial activity. The optimized AgNWs/GO FTCE retained the benefits of the large nano-silver particle surface area when spray-coated on the mask due to its excellent uniformity. Furthermore, the mechanism of microbial inactivation of Ag is also highly correlated with temperature [91]. This self-heating antibacterial mask, which combines stable heat generation performance and good antibacterial activity, not only exhibits strong antibacterial power, but also allows hygienic reuse by effectively removing moisture and bacteria adsorbed on the mask through heat.

## 4. Conclusion

In order to fabricate a high-performance cost-effective FTCE, a AgNWs/GO hybrid film deposited through a simple spray coating process was applied to flexible devices to show the feasibility of the AgNWs/GO electrode. The AgNWs/GO electrode was optimized by controlling the distance, spray speed, compressor pressure and spraying volume of GO solution. In the optimized AgNWs/GO FTCE, GO improved the surface roughness of the AgNWs network and widened the contact area between AgNWs. The optimized AgNWs/GO FTCE showed a high transmittance of 88% at a wavelength of 550 nm and a low sheet resistance of 20 Ohm/square. The flexible TFH with the AgNWs/GO FTCE showed a stable saturation temperature of 119°C when a DC voltage of 5 V was applied; its performance was superior to that of a TFH with a single AgNWs TCE. The flexible EL device with AgNWs/GO FTCE showed better luminance than the EL device with a single AgNWs TCE when the same AC voltage was applied. The facial mask sprayed with the AgNWs/GO FTCE showed stable heat generation based on the excellent flexibility, conductivity, and uniformity of the electrode and showed a bacteriostatic reduction rate of 99.7 against both *Staphylococcus aureus* and *Klebsiella pneumonia*. Based on the excellent electrical, optical, and mechanical properties of the AgNWs/GO FTCE and the successful operation of TFH and EL devices, this study confirmed that the AgNWs/GO hybrid electrode is a promising substitute for conventional ITO electrodes in FTCEs.

## Supplementary Material

Supplemental Material

Supplemental Material

Supplemental Material
