# Supplementary material for: Highly flexible Ag nanowire network covered by a graphene oxide nanosheet for high-performance flexible electronics and anti-bacterial applications
Source: Sci Technol Adv Mater. 2021 Sep 15;22(1):794–807. doi: 10.1080/14686996.2021.1963640 (PMC8451606; doi:10.1080/14686996.2021.1963640)
Supplement: Supplemental Material [file TSTA_A_1963640_SM6825.docx]

**Supplementary information**

Highly flexible Ag nanowire network covered by a graphene oxide nanosheet for high-performance flexible electronics and anti-bacterial applications


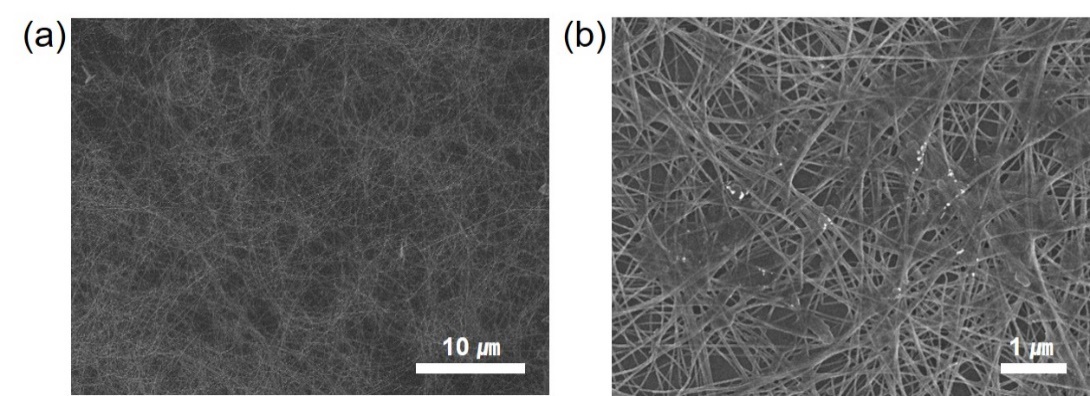


**Figure S1.** Surface FE-SEM images at magnifications of (a) 1,000 x, (b) 10,000 x when 1.0 ml of GO solution was sprayed on AgNWs TCE under the conditions of nozzle-to-substrate distance of 18.0 cm, spray speed of 0.05 ml/s and compressor pressure of 0.20 MPa.


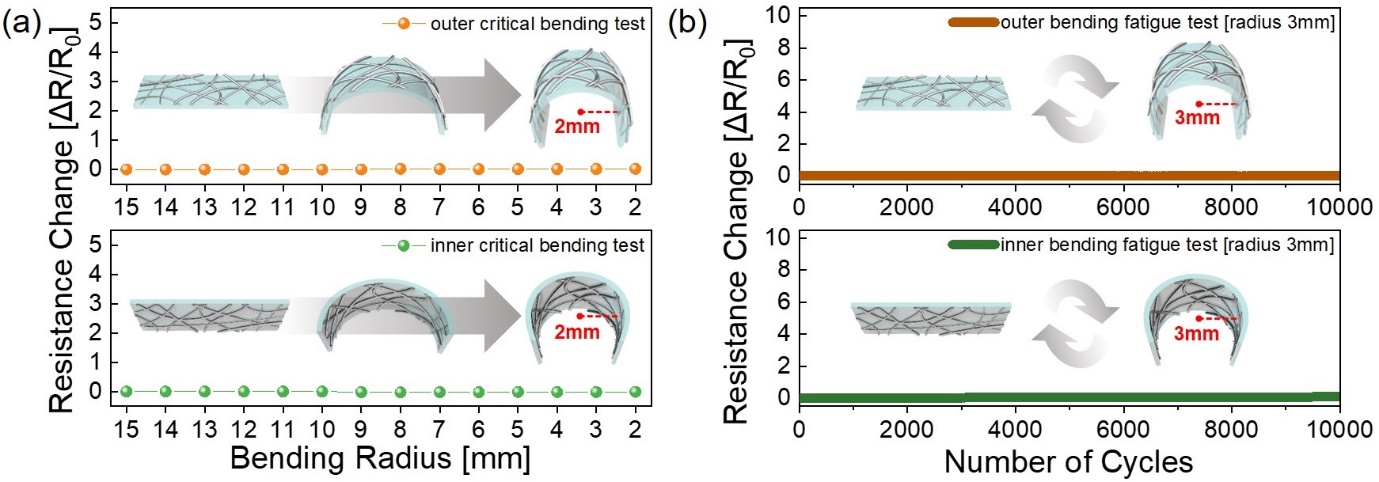


**Figure S2.** (a) Outer/inner bending test of the AgNWs TCE spray coated on PET substrate as a function of bending radius. (b) Outer/inner bending fatigue test of AgNWs TCE repeated 10,000 times at a 3 mm bending radius.


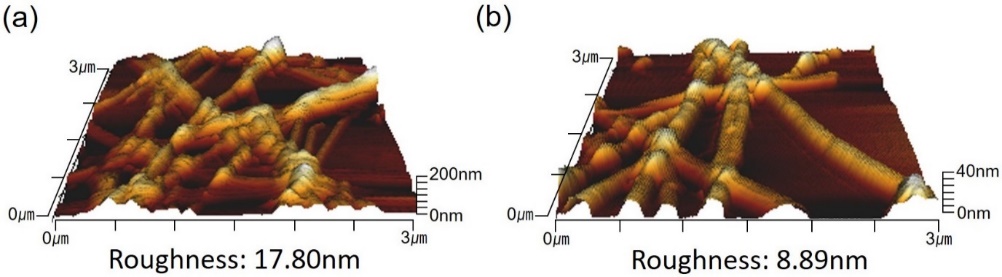


**Figure S3.** AFM images of (a) AgNWs TCE and (b) AgNWs/GO FTCE.


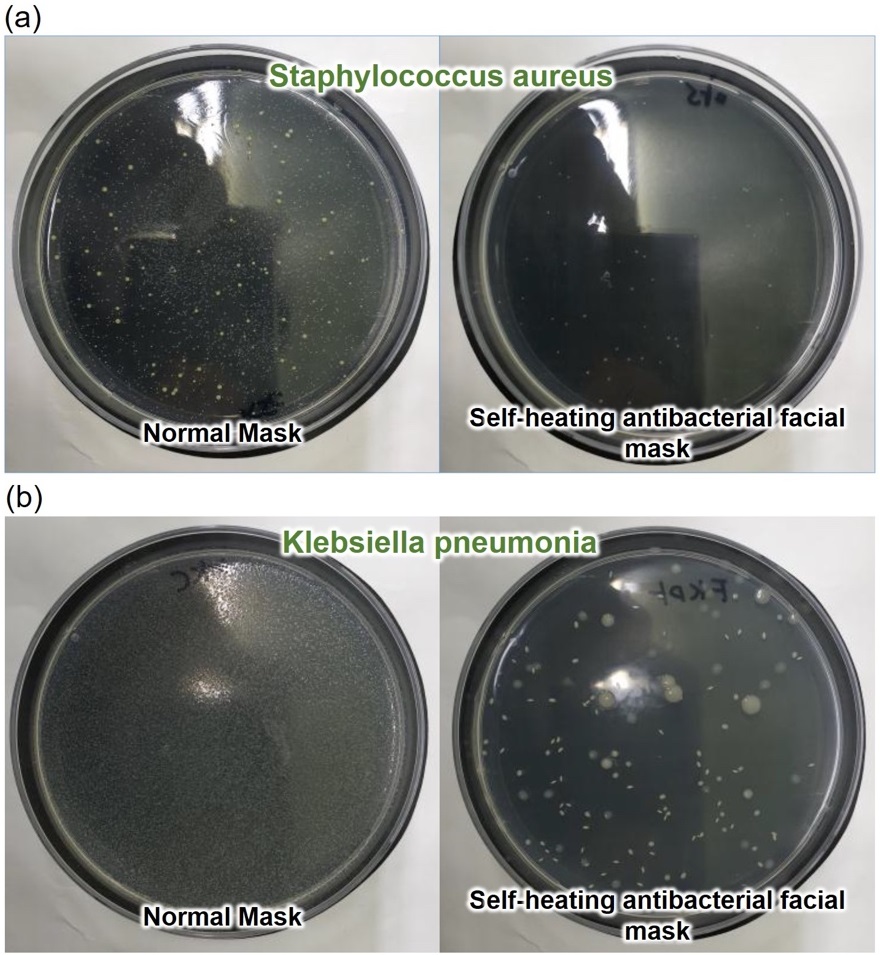


**Figure S4.** (a) Photographs taken 18 hours after the normal mask and self-heating antibacterial facial mask were incubated with the same number of *Staphylococcus aureus* bacteria and (b) photographs taken 18 hours after the masks were incubated with the same number of *Klebsiella pneumonia* bacteria.


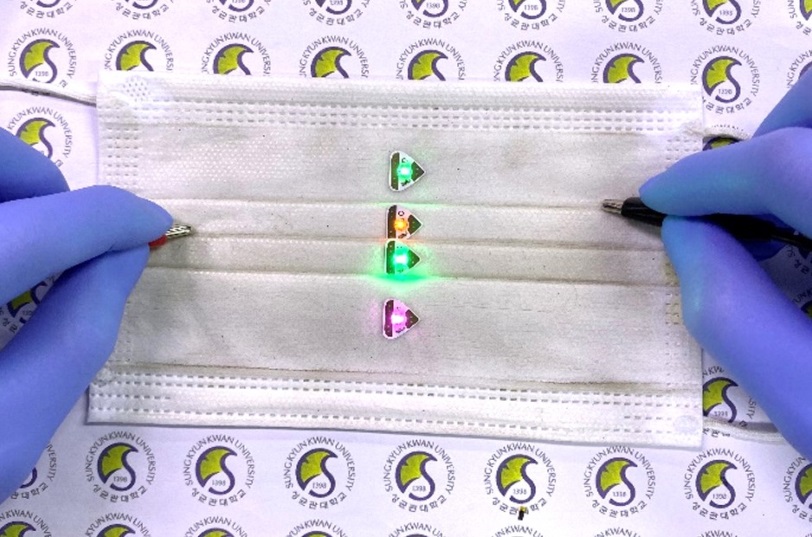


**Figure S5.** Photograph demonstrating the use of the self-heating antibacterial mask as a light-emitting diode interconnector.
